# Histological Features of Delayed Foreign Body Granuloma With Epithelioid Histiocyte Aggregation and Eosinophilic Reaction due to Hyaluronic Acid Injection

**DOI:** 10.1155/crid/5565324

**Published:** 2024-12-19

**Authors:** Michiko Nishimura, Shinnichi Sakamoto, Miyako Hoshino, Yuji Miyazaki, Junichiro Yamamoto, Hideaki Sakashita, Kaoru Kusama, Kentaro Kikuchi

**Affiliations:** ^1^Division of Pathology, Department of Diagnostic and Therapeutic Sciences, Meikai University School of Dentistry, 1-1 Keyakidai, Sakado, Saitama 350-0283, Japan; ^2^Division of Basic Biology, Department of Oral Biology and Tissue Engineering, Meikai University School of Dentistry, 1-1 Keyakidai, Sakado, Saitama 350-0283, Japan; ^3^Department of Oral and Maxillofacial Surgery, Abiko Seijinkai Hospital, 1300 Shibasaki, Abiko, Chiba 270-117, Japan

**Keywords:** delayed foreign body granuloma, eosinophilic infiltration, granulomatous reaction, hyaluronic acid, pseudoduct-like structure

## Abstract

**Background:** Dermal fillers such as hyaluronic acid (HA) have been widely used in recent years as a less surgically invasive cosmetic treatment. Although delayed foreign body granuloma may occur as a rare adverse reaction after the procedure, detailed histological reports are still limited. When occurring on the buccal mucosa of the oral cavity, the histopathology may resemble some lesions of minor salivary gland origin due to the material properties of HA. Here we report a delayed foreign body granuloma associated with HA showing eosinophilic infiltration of the buccal mucosa, with characteristic histological and immunohistological features.

**Case Presentation:** A 61-year-old woman presented with swelling and a burning sensation in the right buccal mucosa. On initial examination, a 25 × 20-mm mass was palpated on the anterior margin of the right masseter muscle. Examination of a biopsy specimen revealed multiple pseudoduct-like structures containing mucoid substance within the mucosal lamina propria. The mucoid substance was positively stained with Alcian blue (AB) and surrounded by CD68-positive epithelioid cells and multinucleated giant cells. Many histiocytes had infiltrated into the surrounding area, and numerous eosinophilic infiltrates were also evident. After a review of the patient's history, a diagnosis of delayed foreign body granuloma associated with HA injection was made.

**Conclusion:** We have reported a case of delayed foreign body granuloma with eosinophilic and histiocytic infiltration following injection of HA. It was suggested that the marked eosinophilic infiltration around macrophages was due to not only an allergic reaction, but also in part to increased macrophage aggregation.

## 1. Introduction

Injection of dermal fillers such as hyaluronic acid (HA) has become common in recent years as a less surgically invasive cosmetic treatment. However, the widespread use of this procedure has led to occasional adverse complications, such as infections and foreign body granulomas. In comparison to other fillers, HA fillers are relatively stable and have good biocompatibility [1]. Adverse reactions to HA mostly occur within the first few days after the procedure, and are usually characterized as localized redness at the injection site, eruptions, oedema and pruritus. The incidence of delayed-type hypersensitivity (Type IV hypersensitivity) is low, being less than 1% in most recent reports [2, 3]. However, the possibility of delayed-type hypersensitivity with eosinophil infiltration should be borne in mind, as the number of such procedures has increased in recent years. Another important adverse reaction is necrosis due to embolism or compression after HA injection [4]. As dermal fillers such as HA are essentially intended as a form of cosmetic treatment, adverse complications such as unintended granuloma formation should naturally be avoided. It is therefore important to be aware that such adverse complications can occur. At present, given the infrequent nature of delayed granulomas, reports detailing their histopathological features are still limited [5]. Here we describe the histological and immunohistological features of delayed foreign body granulomas associated with HA injection and discuss the pathogenesis, along with diagnostic considerations in cases affecting the buccal mucosa, including the minor salivary glands.

## 2. Case Presentation

**Figure 1:**
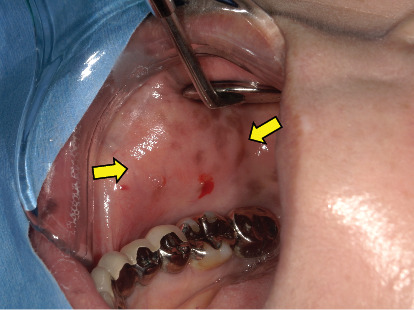
Clinical photograph of foreign body granuloma due to HA injection. A slightly flattened, large nodular lesion on the right cheek (arrow).

**Figure 2:**
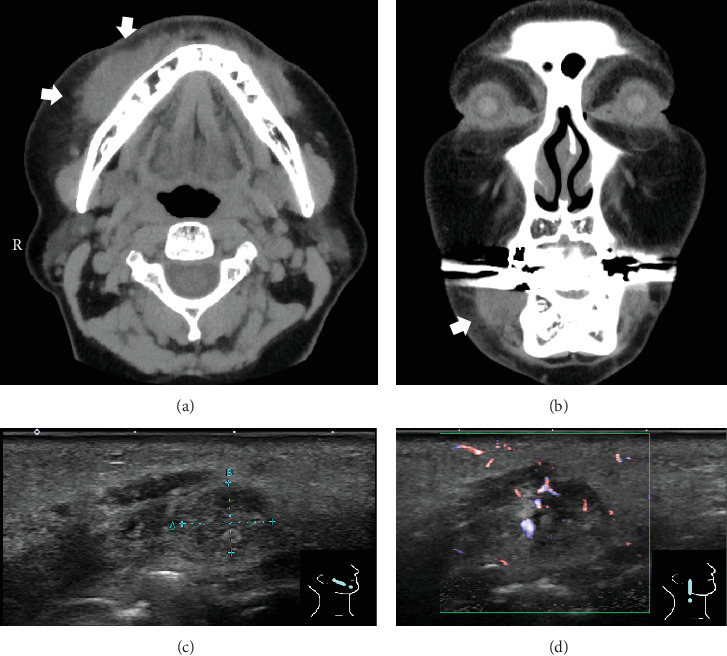
Clinical images of the patient at the first visit. (a, b) Computed tomography of the neck. A lesion equal in density to muscle was found under the right subcutaneous mandible (arrow). The tumour measured approximately 28 × 15 mm in thickness. There was an increased density of surrounding fatty tissue, suggesting an inflammatory mass ((a) axial image; (b) coronal image). (c, d) Findings of ultrasound examination. A 9 × 7-mm lesion was detected in the subcutaneous tissue of the right buccal area, continuous with the muscular layer. The border was slightly indistinct and oval in shape with heterogeneous hypoechoic internalization. Doppler imaging shows a penetrating vascular form (arterial waveform).

A 61-year-old Japanese female patient presented to the oral and maxillofacial surgery department of a local hospital with complaints of swelling and burning in the right cheek, associated with a small mass in the left buccal mucosa. There had been no particular pain or other symptoms until presentation. At the initial examination, a focally localized flat submucosal nodule was palpated at the anterior margin of the masseter muscle in the right buccal region. The nodule was mobile, without tenderness, and no surrounding induration was palpable (Figure 1). There were no obviously enlarged cervical lymph nodes bilaterally. A review of the patient's medical history revealed that she had undergone plastic surgery involving HA injections in both cheeks one year earlier. Computed tomography (CT) demonstrated a 28 × 15-mm subcutaneous mass in the right buccal region, with a density equal to that of muscle (Figures 2(a) and 2(b)). CT revealed a high-density region in the buccal fat layer of the right buccal soft tissue, suggesting an inflammatory mass lesion.

Echographic examination demonstrated a lesion in the right buccal area with slightly unclear borders, with oval-like, internally hypoechoic heterogeneity. The nodule had an internal doppler signal and was approximately 9 × 7 mm in size, extending from the subcutaneous tissue to near the muscle (Figures 2(c) and 2(d)).

**Figure 3:**
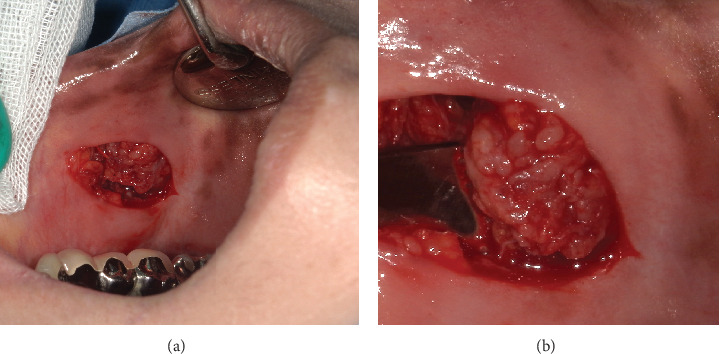
Gross photographs of foreign body granuloma due to HA injection. (a, b) Intraoperative clinical features of the lesion. The lesion was manifested as a cluster of individual small granular masses.

An excisional biopsy was performed as there had been no significant change, except for a slight reduction in size after administration of amoxicillin capsules one week before surgery. Surgery was performed using an intraoral approach. After local anaesthesia, a transverse incision approximately 30 mm long was made directly over the mass, which was removed en bloc by detaching it from the surrounding tissue. The submucosal lesion was found to contain a small, translucent, jelly-like granular body (Figures 3(a) and 3(b)). The mass showed no capsule formation, and was difficult to remove as it adhered to the surrounding tissue. After completion of the surgical procedure, the wound was closed. Subsequent wound healing was excellent, and the patient is currently being followed up with no evidence of a recurrence or reswelling of the mass.

## 3. Histopathology

**Figure 4:**
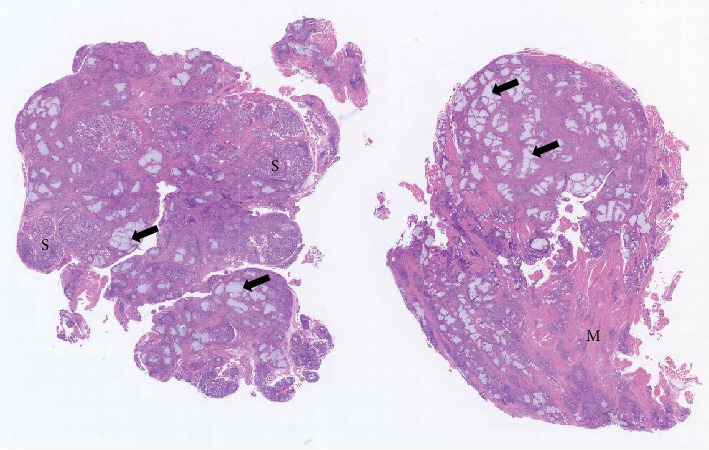
Scanning view of the excisional biopsy specimen of nodular granuloma containing HA. Granulation tissue engulfs the surrounding minor salivary glands (S) and muscle tissue (M) (arrow, hyaluronic acid).

**Figure 5:**
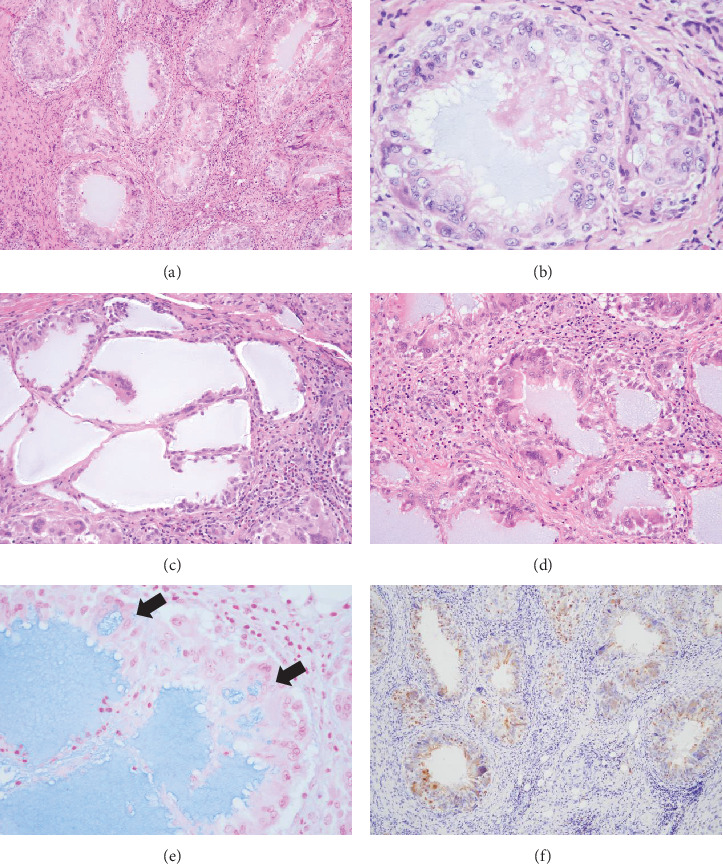
Histopathology showing a delayed granulomatous reaction associated with HA injection. (a) Aggregation of numerous multinucleated giant cells and epithelioid cells showing a pseudoduct-like structure within the mucosal lamina propria. The inner spaces are filled with a lightly basophilic myxoid substance. (b) Macrophage aggregates resembling tumour nests with mucin-like components were occasionally seen. (c, d) HA-derived substances show similar staining to mucin-like substances in pool-like structures of various sizes. (e) The internal HA is positive for Alcian blue staining. The uptake of the HA substance into the cytoplasm of macrophages is also evident (arrow). (f) Immunohistochemistry showing CD68-positive multinucleated giant cells and epithelioid cells surrounding a pseudoduct-like structure. (a–d) H&E; (e) Alcian blue stain; and (f) CD68.

**Figure 6:**
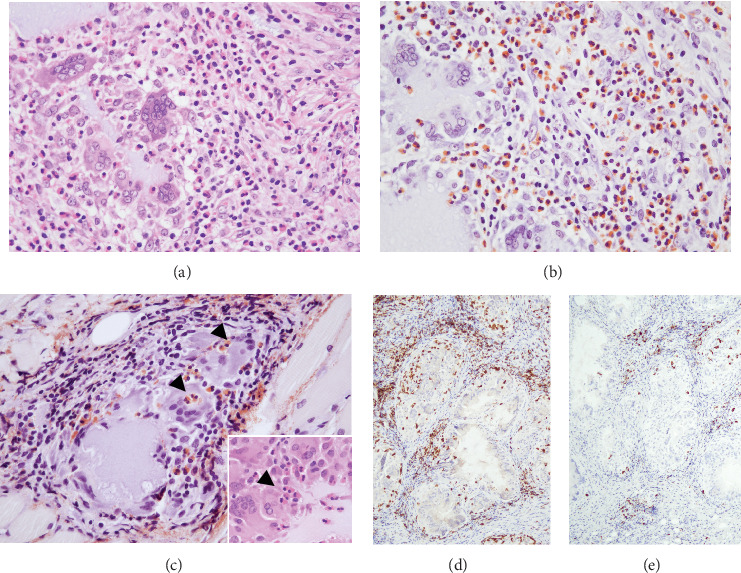
Characteristic dense eosinophilic infiltrate and inflammatory reaction associated with HA. (a) Prominent eosinophilic infiltrate surrounding macrophages within the foreign body granuloma. (b) DFS staining reveals a marked eosinophilic infiltrate. (c) Significant eosinophil degranulation and eosinophil uptake into macrophages is evident (arrowhead) (inset, H&E). (d, e) At the periphery of the macrophages, there is also a chronic inflammatory infiltration of mainly CD3- and CD8-positive cells. (a) H&E; (b, c) DFS stain; (d) CD3; and (e) CD8.

Histopathologically, the biopsy specimen showed nodular infiltration of the deep submucosal tissue, including the muscle and minor salivary gland (Figure 4), and contained numerous pseudoglandular spaces or small pool-like structures (Figures 5(a) and 5(b)). The interior of the small pool-like structures contained a basophilic material, HA gel, with a mucinous appearance (Figures 5(c) and 5(d)). Characteristic aggregation of multinucleated giant cells around small pool-like structures was diagnostically significant (Figure 5(d)). An Alcian blue (AB)–positive mucin-like substance had been phagocytized by CD68-positive epithelioid-like cells and giant cells (Figures 5(e) and 5(f)). Some of the clusters of aggregated macrophages had tumour nest-like structures, including those that closely resembled mucous cells (Figures 5(b) and 5(e)). In addition, massive peripheral infiltrates of eosinophils were observed around the macrophages (Figure 6(a)), showing obvious eosinophil degranulation and subsequent macrophage phagocytosis on DFS (direct fast scarlet) staining (Figures 6(b) and 6(c)). CD3- and CD8-positive lymphocytes were also well infiltrated at the periphery of the macrophages (Figures 6(d) and 6(e)).

The biopsy results were suggestive of a foreign body granuloma composed of epithelioid-like cells and giant cell macrophages. Histologically, multiple pseudoglandular spaces containing AB-positive HA filler were observed. The HA consisted of sulphated acid mucin-containing material and a marked eosinophilic infiltrate was also observed around the HA. The final pathological diagnosis was delayed foreign body granuloma associated with HA injection.

## 4. Discussion

In recent years, there has been an increase in the use of less surgically invasive cosmetic treatments in the orofacial area. Fiuza et al. have reported the formation of foreign body granulomas and changes in the appearance of the upper lip due to long-term complications attributable to the remnant of nonbioresorbable fillers, and permanent fillers such as polydimethylsiloxane, also known as liquid silicone. In their paper, Fiuza et al. state that the ideal biomaterial should be safe and stable after application, compatible with the local tissue, not migrate from the implantation site, and pliable and maintain volume without being absorbed [6]. To reduce the risk of adverse events, it is important for professionals to understand the properties of biomaterials as well as possible tissue reactions.

HA is a natural polysaccharide found in the body, and is therefore more biocompatible and less likely to cause allergic reactions than other materials such as silicone or collagen. Although HA injection often causes initial symptoms of general inflammation such as redness, swelling and internal bleeding, foreign body granuloma as a delayed hypersensitivity reaction is very rare [7, 8]. In patients receiving HA injections, Hwang et al. and Friedman et al. reported that the risk of delayed hypersensitivity reactions was around 0.4% and 0.02%, respectively [9, 10]. As it has also been reported that granulomas form in individuals with a history of repeated HA injections [11], a full assessment of any history of previous procedures and the composition of the material used is very important for understanding the etiology. Necrosis and embolization have also been reported as other serious complications [4] due to perivascular compression and vascular occlusion (embolization) caused by overcorrection of the HA. Considering that HA is used cosmetically, efforts should be made to avoid serious complications.

Factors contributing to the formation of foreign body granulomas due to HA filler include the composition of the injected material, such as impurities arising from bacterial fermentation during HA purification; the shape and size of the injected particles; or host environmental factors such as infection, trauma and immunity [10, 11]. In the present case, delayed granuloma formation was observed approximately one year after the procedure. A left–right difference in the size of the lesion was also observed. There had been bilateral injections of HA into the cheek, with no significant symptoms until the patient became aware of a mass in the right cheek area. In cases of granuloma formation due to inflammatory reactions during the process of HA absorption, it is considered important to monitor any progressive changes, taking into account host factors, the injection volume, and the specific properties of the HA filler.

Histologically, the granulation tissue in the present case showed marked degranulation due to eosinophil infiltration and foreign body phagocytosis (Figures 6(a), 6(b), and 6(c)). GM-CSF (granulocyte-macrophage colony-stimulating factor) is a haematopoietic growth factor produced by macrophages and T cells that, together with IL-3 and IL-5, induces the differentiation of bone marrow progenitor cells into eosinophils and the differentiation and proliferation of macrophages [12, 13]. In the present case also, it is suggested that increased production of GM-CSF by macrophages may have influenced the increase in eosinophils. On the other hand, an increase in eosinophils resulting from the inflammatory response could have triggered macrophage activation. A similar pattern was observed in studies by Mu et al. and Okada et al., where macrophage clusters surrounding HA were evident around a pronounced eosinophilic infiltrate [5, 7]. The prominent appearance of regional eosinophils suggests that they may influence the phagocytic response of histiocytes. Although the mechanism is unclear, allergic reactive eosinophilic infiltration associated with HA degradation and cytokine-induced reactions such as GM-CSF due to increased histiocytes are suspected. Factors contributing to the systemic increase in eosinophils include an endogenous increase due to genetic mutations in haematopoietic stem cells and myeloid cells, and an exogenous increase due to cytokine secretion from T cells and tumour cells. GM-CSF is found at high levels in joints affected by rheumatoid arthritis, and granulomatous reactions have also been reported following the use of antirheumatic drugs such as leflunomide [11, 14]. The present patient had no systemic abnormalities or significant medical history, and all inflammatory markers and laboratory data, including elevated eosinophils, were normal.

Imbalances in the regional immune-inflammatory cascade are thought to be a primary factor in the formation of granulomas with eosinophilic infiltrates.

This patient had not been treated with hyaluronidase. Hyaluronidase is useful for preventing serious complications such as adverse reactions after HA injection, especially ischemia and necrosis due to overfilling. In a study investigating the ability of hyaluronidase to degrade HA, 0.3 mL of HA was directly immersed in hyaluronidase solution in a test tube and was almost completely hydrolysed in 2 h [4]. A 60% reduction was also observed in an experimental embolization case where a vein was filled with HA. If, as in this case, HA remains a foreign body granuloma, a reduction of HA filler due to hyaluronidase may reduce the occurrence of macrophages and suppress any excessive foreign body reaction.

Histologically, the HA fillers were positively stained with AB and showed a characteristic tubular or puddle-like structure (Figures 5(a), 5(b), 5(d), and 5(e)). AB staining is useful for distinguishing artificial materials other than HA, as AB does not stain any other artificial fillers such as collagen or silicone-derived materials. Foreign body granulomas in the orofacial region can be caused by long-term use of permanent fillers such as polydimethylsiloxane (liquid silicone) [6] or by other artificial materials.

AB staining was also very useful in the present case and provided a basis for confirming the HA-derived filler material (Figure 5(e)). In addition, some of the clusters of HA-containing macrophages and epithelioid-like cells morphologically resembled tumour nests with mucus-like cells (Figures 5(b) and 5(e)). HA and mucus cells are generally positive for AB staining. Mucus and macrophages are often observed in aspirate cytology specimens of cystic low-grade mucoepidermoid carcinoma [15]. Therefore, when HA-derived material is very small in size and occurs in an area where minor salivary glands are present, such as the buccal mucosa in the present case, care should be taken not to confuse it with low-grade mucoepidermoid carcinoma or other mucin-producing minor salivary gland tumours.

The accessory lobe of the parotid gland (APG) is an ectopic salivary gland in the middle of the cheek, present in 21–69% of the population [16]. Tumours occurring in the APG account for only 1% of all parotid gland tumours, but are associated with a higher incidence of malignancy than those in the main parotid gland [16, 17]. It is therefore very important to differentiate a mass in the buccal mucosa from other malignant tumours, such as salivary gland tumours of APG or minor salivary gland origin and malignant lymphomas. In the present case, the mass in the minor salivary gland region was located anteriorly, lower and further away from the APG, as shown by CT (Figures 2(a) and 2(b)). The CT image of the foreign body granuloma suggested an inflammatory mass with homogeneous density, lacking the heterogeneity characteristic of a minor salivary gland tumour. Even if resected as a benign lesion, it must be differentiated from a low-grade malignant tumour, and in this case, the histology showed a mucus-like substance and some mucus-like cells within the mass, which required differentiation from a mucin-producing minor salivary gland tumour.

HA injection is widely recognized around the world as a relatively safe cosmetic procedure with high biocompatibility, and its use has thus spread rapidly in recent years. However, because of the possibility of delayed complications such as granuloma formation [18], patients should be fully informed about the possibility of granuloma formation beforehand. In addition, as this cosmetic procedure has been used increasingly in recent years, any history of HA injection should be carefully considered in the histopathologic diagnosis.

## 5. Conclusion

We have reported a case of a delayed foreign body reaction granuloma with eosinophilic and histiocytic infiltration following injection of HA.

## Data Availability

The imaging data used to support the findings of this study are included in the article.

## Nomenclature

HA: hyaluronic acid
CT: computed tomography
AB: Alcian blue
DFS: direct fast scarlet
GM-CSF: granulocyte-macrophage colony-stimulating factor
